# Accumulation of high OPDA level correlates with reduced ROS and elevated GSH benefiting white cell survival in variegated leaves

**DOI:** 10.1038/srep44158

**Published:** 2017-03-09

**Authors:** Ying-Hsuan Sun, Chiu-Yueh Hung, Jie Qiu, Jianjun Chen, Farooqahmed S. Kittur, Carla E. Oldham, Richard J. Henny, Kent O. Burkey, Longjiang Fan, Jiahua Xie

**Affiliations:** 1 Department of Forestry, National Chung Hsing University, Taichung 402, Taiwan; 2 Department of Pharmaceutical Sciences, Biomanufacturing Research Institute & Technology Enterprise, North Carolina Central University, Durham, NC 27707, USA; 3 Department of Agronomy, Zhejiang University, Hangzhou 310029, China; 4 Environmental Horticulture Department and Mid-Florida Research and Education Center, University of Florida, Apopka, FL 32703, USA; 5 USDA-ARS Plant Science Research Unit and Department of Crop Science, North Carolina State University, Raleigh, NC 27695, USA

## Abstract

Variegated ‘Marble Queen’ (*Epipremnum aureum*) plant has white (VMW) and green (VMG) sectors within the same leaf. The white sector cells containing undifferentiated chloroplasts are viable, but the underlying mechanism for their survival and whether these white cells would use any metabolites as signal molecules to communicate with the nucleus for maintaining their viability remain unclear. We analyzed and compared phytohormone levels with their precursors produced in chloroplasts between VMW and VMG, and further compared their transcriptomes to understand the consequences related to the observed elevated 12-oxo phytodienoic acid (OPDA), which was 9-fold higher in VMW than VMG. Transcriptomic study showed that a large group of OPDA-responsive genes (ORGs) were differentially expressed in VMW, including stress-related transcription factors and genes for reactive oxygen species (ROS) scavengers, DNA replication and repair, and protein chaperones. Induced expression of these ORGs could be verified in OPDA-treated green plants. Reduced level of ROS and higher levels of glutathione in VMW were further confirmed. Our results suggest that elevated OPDA or its related compounds are recruited by white cells as a signaling molecule(s) to up-regulate stress and scavenging activity related genes that leads to reduced ROS levels and provides survival advantages to the white cells.

The chloroplast is not only a photosynthetic center for energy transfer but also an essential organelle for generating important cellular molecules such as amino acids, fatty acids and carbohydrates as well as precursors of vitamins and phytohormones^1^. In order to support plant cell survival and function, chloroplasts can sense and mitigate biotic and abiotic stresses using their generated metabolites as signals to regulate neighboring subcellular organelles including the nucleus^1^. These signals could potentially modulate the expression of nuclear genes via metabolic retrograde signaling pathways^2^. The metabolites produced in the chloroplast that function as signaling molecules including reactive oxygen species (ROS)^3^,^4^, sugars^5^,^6^, 12-oxo phytodienoic acid (OPDA) of the jasmonic acid (JA) biosynthesis pathway^7^,^8^, tetrapyrrole intermediates of chlorophyll biosynthesis pathway^9^ and methylerythritol cyclodiphosphate of the methylerythritol phosphate (MEP) pathway^10^ as well as phytohormones derived from chloroplast-produced precursors, such as gibberellin (GA), abscisic acid (ABA), salicylic acid (SA) and JA^11^,^12^.

Variegated plants, such as Arabidopsis *im, var1* and *var2* mutants, have been widely used for studying chloroplast development and cell communication between green and color defective sectors^13^,^14^,^15^,^16^. Studies of color defective cells in these mutants have confirmed that all yellow/white cells were viable despite the fact that their chloroplasts were abnormal^17^,^18^. However, little is known about how these white/yellow cells maintain their growth and development since they have reduced metabolic capacities^19^ and are more prone to biotic and abiotic stresses^20^,^21^ than green cells. Transcriptome analysis of green and white sectors of *im* and *var2* in comparison to wild type suggested an enhanced sugar catabolism and transport in green sectors for supporting the adjacent white sectors^15^,^16^. However, whether these color defective cells would use any metabolites as signal molecules similar to those green cells used to communicate with the nucleus for maintaining their viability remains unclear.

Among the chloroplast generated metabolites that are used as retrograde signaling molecules, ROS is considered to be an important player in color defective cells. However, it does not appear to be the case in *var2* as its green sectors accumulated higher levels of ROS and experienced more photo-oxidative stress under normal light conditions than white sectors^16^,^17^. No reasonable explanation thus far has been given to this phenomenon. Since the expression levels of genes involved in scavenging activity were significantly increased whereas ROS levels were dramatically reduced in *var2* white sector^16^, the question of whether any accumulated metabolite in white cells that plays a role to regulate scavenging activity and to further maintain low levels of ROS for survival is still unanswered. From the study of Arabidopsis *flu* mutant, OPDA appeared to be a favorable candidate for reducing ROS via induced scavenging activity^22^. OPDA, a JA precursor, has been implicated as a signal molecule for stress tolerance^8^,^23^. Its levels were induced during wounding and insect herbivory^24^,^25^. Recent studies also showed OPDA regulates a unique subset of responsive genes distinct from JA specific responsive genes^7^, as well as has its own receptor CYP20-3^8^, indicating that OPDA plays a unique role in stress tolerance. In contrast to this hypothesis, recent studies of two JA biosynthesis mutants, *cpm2* and *hebiba*, show less accumulation of OPDA associated with less severe symptoms than wild type in response to salt stress followed by ROS accumulation^26^, implying increased OPDA at high concentration contributes to cell damage. Whether OPDA plays a role of dampening ROS-induced cytotoxicity or enhancing cell damage requires further investigation.

Two varieties of *Epipremnum aureum*, white-variegated ‘Marble Queen’ and yellow-variegated ‘Golden Pothos’, were selected to investigate above questions. Cells in both yellow and white tissues from variegated leaves are viable and have the ability to regenerate new yellow and white plantlets, respectively^27^,^28^. PCR analysis of chloroplast genes in both regenerated pale yellow and white plants showed that there was no difference in chloroplast genome integrity compared to green plants^28^,^29^. In addition, the total protein profiles of regenerated pale yellow and white plants in comparison to the green plants were different in pattern but not in quantity and complexity^27^,^28^,^29^. These results further support that yellow and white cells are viable. Furthermore, compared to variegated Arabidopsis mutants, variegated Epipremnum plants have greater biomass, which makes metabolite analysis more possible. Recently, we also established the transcriptome of ‘Marble Queen’^30^, which could be used for molecular study.

In the present study, we used ‘Marble Queen’ with distinct large areas of VMW and VMG sectors within the same leaf (Fig. 1a) as materials to compare both metabolites and transcriptome profiles between the two sectors. Because phytohormones are retrograde signaling molecules that play important functions of modulating nuclear gene expressions to cope with environmental changes, we first examined the levels of those chloroplast-related phytohormones including SA, ABA, and jasmonates including JA, OPDA and JA conjugate, jasmonoyl-L-isoleucine (JA-Ile). GAs were not measured because the GA biosynthetic gene *EaGA3ox1* is silenced in Epipremnum variety ‘Jade’, a ‘Marble Queen’ green reversion, and none or only trace amounts of GAs were detected previously^30^. We found that ‘Marble Queen’ white sectors accumulated ~9-fold greater of OPDA compared to green sectors, whereas JA and JA-Ile were not detectable in either sector. To understand the consequences of elevated OPDA in VMW on nuclear gene expressions, RNA-Seq analysis was employed to identify differentially expressed genes (DEGs). Among them, there are a large group of OPDA-responsive genes responsible for scavenging activities, maintaining DNA function and protein chaperones. They were uniquely up-regulated in VMW, and their induced expressions were further verified in OPDA-treated green plants. These genes should play important roles to protect VMW cells from oxidative stress and their differential expressions very likely relate to the elevated levels of OPDA in VMW.

## Results

### VMW cells have smaller chloroplasts with loose membranes

**Figure 1:**
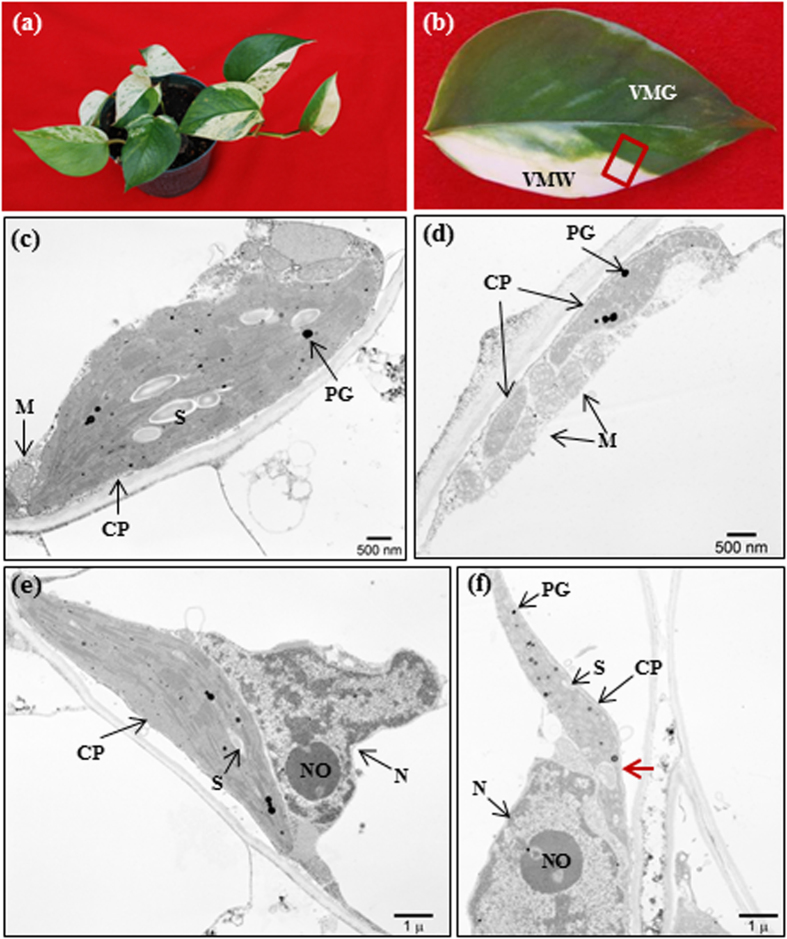
Variegated ‘Marble Queen’ plant and its leaf TEM observation of VMG and VMW. (**a**) A variegated ‘Marble Queen’ plant. (**b**) A variegated leaf with a large area of white sector. Region marked in red was used for TEM analysis in VMG (**c**,**e**) and VMW (**d**,**f**). CP = chloroplasts; M = mitochondria; N = nucleus; NO = nucleolus; PG = plastoglobules; S = starch granule. The red arrows indicate loose plastid membrane with inclusion of mitochondria.

Our previous studies showed that cells in yellow sectors of ‘Golden Pothos’ have few chloroplasts^29^ and are viable^27^. Similarly, ‘Marble Queen’ white sector cells are also viable^28^. To correlate the white cell viability of ‘Marble Queen’ with cell integrity, a leaf with distinctive VMW and VMG sectors (Fig. 1b) was chosen to examine the subcellular organelles with TEM. Compared to the green cells (Fig. 1c,e), the white cells had few much smaller chloroplasts with thylakoid membranes not well-organized to form typical grana (Fig. 1d,f). The outer and inner membranes which define the structure of the chloroplasts of VMW appeared to be loosening that could be the reason for the observed intrusion of a neighboring organelle (Fig. 1f). Both plastoglobules (lipid storage bodies) and starch granules were visible in some of these smaller chloroplasts (Fig. 1d,f), suggesting certain catabolism of lipid and carbohydrates was still active. The nucleus and mitochondria appeared normal morphologically (Fig. 1d,f), consistent with observed results from regenerated pale yellow plants of ‘Golden Pothos’^27^. Moreover, the sizes of nucleoli were also similar between green and white cells (Fig. 1e,f), which is different from the report of enlarged nucleoli found in *var2* white cells^18^. Overall, chloroplasts found in VMW are closer in appearance to those observed in photoautotrophic hairy root suspension cultures^31^,^32^ which are capable of producing secondary metabolites^33^.

### VMW cells have reduced pigment contents

The disappearance of grana and presence of the unstructured thylakoid membrane observed in VMW are likely the result of the absence of pigments. Pigments are major components and essential for the assembly of light-harvesting complex^34^, allowing Lhcb protein to attract the opposite side of thylakoid membranes to form grana^35^. Thus, the levels of Chls and carotenoids, the two major photosynthetic pigments, were determined. The results showed that VMW contained 33-fold less Chl*a*, 25-fold less Chl*b* and 10-fold less carotenoids than those in VMG (Supplementary Fig. S1a), confirming the deficiency of these pigments in color defective VMW.

### No deficiency of major elements in VMW Cells

Nutrient deficiency would cause leaf chlorosis and growth reduction^36^. To determine if any elemental deficiency was related to VMW formation, a total of 11 elements were compared between VMW and VMG. None were found to be less in VMW (Supplementary Fig. S1b). Additionally, five elements, P, K, S, Fe and Zn, were significantly higher in VMW than in VMG (above 10%) with Zn increased ~25%. This result shows that the growth of VMW cells in this study was not impacted by elemental deficiency.

### VMW cells have elevated levels of OPDA

**Figure 2:**
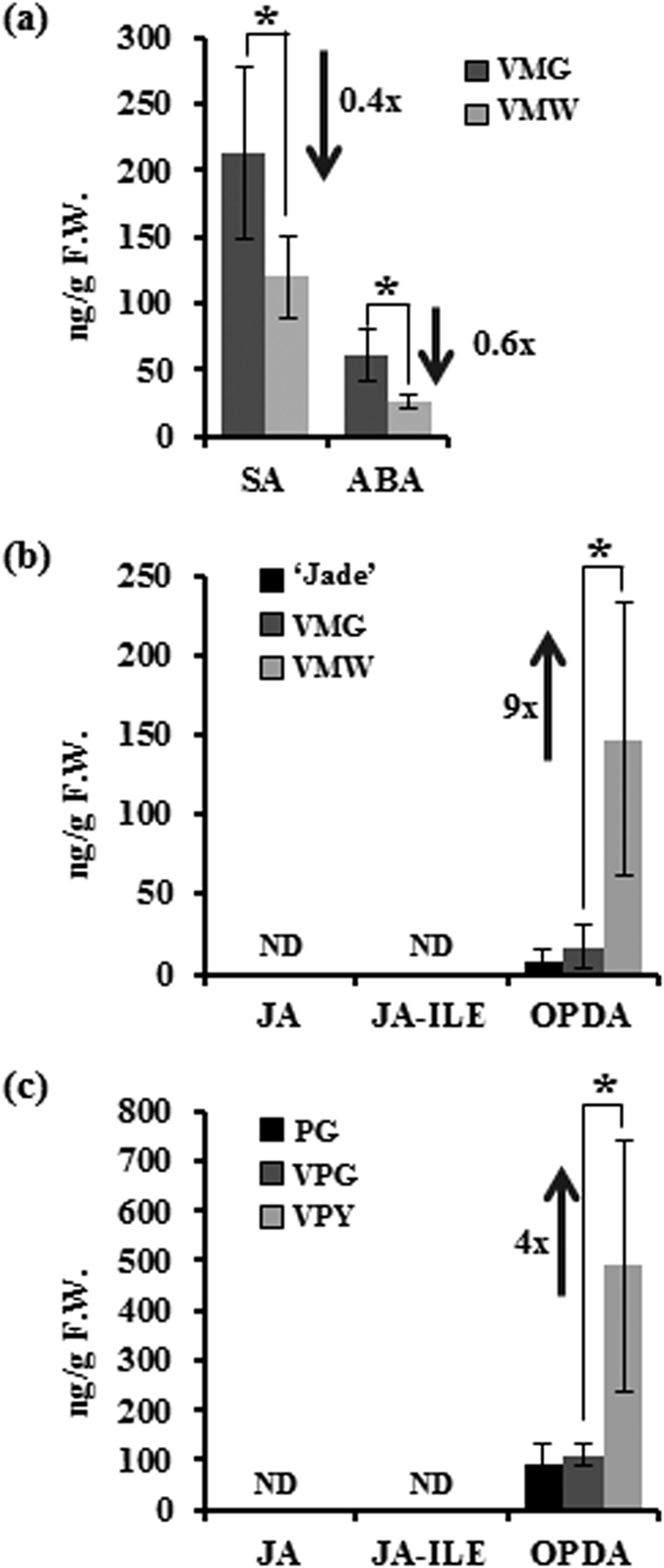
Analysis of phytohormone contents. An equal amount of leaf sectors from ‘Marble Queen’ (**a**,**b**) and ‘Golden Pothos’ (**c**) as well as pure green plants ‘Jade’ and PG, respectively, were used for measuring. Data represents an average from three independent pairs of sectors ± SD. For pure green plants, data represents an average of three independent leaves ± SD. The arrow represents an increase (↑) or decrease (↓) in fold (x). F.W., fresh weight; ND, not detectable; *P < 0.05.

VMW cells with few small chloroplasts may produce lower quantities of metabolites. Among chloroplast-produced metabolites, acidic phytohormones are important for plant cell growth and development as well as biotic and abiotic stress responses. It has been reported that in variegated leaves certain metabolites in white sectors can be possibly compensated from green sectors of Arabidopsis^15^,^16^, but uncertainty remains about the accumulation levels of these phytohormones in VMW. Thus, we analyzed them and found that SA and ABA were reduced by 40% and 60% in VMW compared to VMG, respectively (Fig. 2a). Both JA and JA-Ile were not detectable in either VMW or VMG while OPDA, a JA precursor, was ~9-fold higher in VMW than VMG (Fig. 2b). To verify if OPDA is low only in VMG, the OPDA level was also determined for the green plant ‘Jade’. ‘Jade’ and VMG have similar low levels of OPDA while JA and JA-Ile were also undetectable in ‘Jade’ (Fig. 2b).

Such high OPDA levels in VMW and undetectable JA in both VMW and VMG were unexpected. Any less-produced or absent metabolites in VMW could be compensated from the adjacent VMG only up to equilibrium levels between two sectors unless there is an active transporter that can cause the hyper-accumulation in VMW. It was reported that both JA and OPDA are present at relatively low basal levels and can be transiently induced after wounding and pathogen attack^24^,^25^. In Arabidopsis, the basal levels of JA and OPDA ranged from 30 to 40 ng g^−1^ and 270 to 400 ng g^−1^ fresh weight, respectively, whereas JA and OPDA are known to increase ~40- and ~2.5-fold, respectively 90 min after wounding^24^. In ‘Marble Queen’, undetectable levels of JA in both sectors imply that there was possibly a defect in the conversion of OPDA to JA. However, constantly higher levels of OPDA in VMW than VMG might suggest that OPDA plays an important role in dealing with the special physiological status.

To verify whether elevated OPDA in VMW is a unique phenomenon in ‘Marble Queen’ or occurs as a general phenomenon in other variegated Epipremnum plants, OPDA as well as JA contents of yellow (VPY) and green (VPG) sectors from variegated ‘Golden Pothos’ leaves were also measured. The PGs regenerated from green leaf sectors of ‘Golden Pothos’^27^ were included as a control. The VPG and PG had low levels of OPDA, but VPY had 4.4-fold higher OPDA than VPG (Fig. 2c). Similar to ‘Marble Queen’, all three types of tissues (PG, VPG, and VPY) from ‘Golden Pothos’ had undetectable JA and JA-Ile (Fig. 2c). These results indicate that constantly elevated levels of OPDA are common in color defective sectors.

### A large number of genes were differentially expressed in VMW

**Figure 3:**
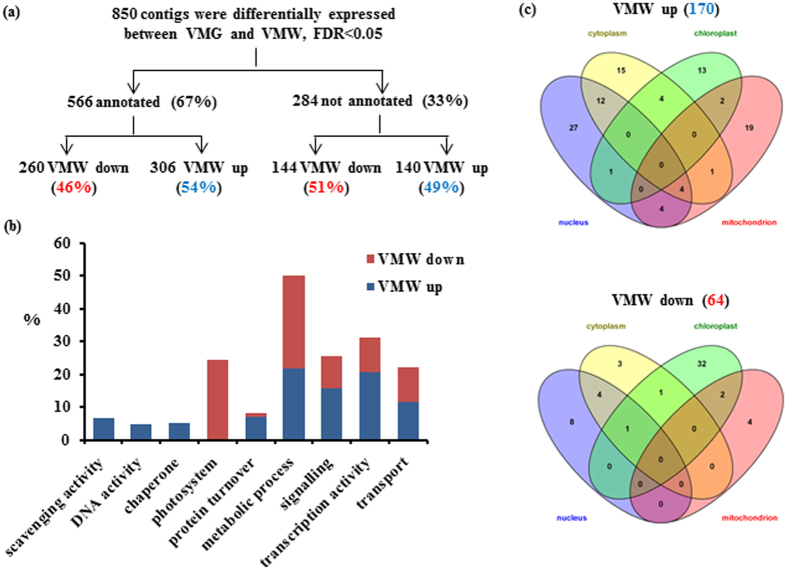
Comparative DEG analysis of VMG and VMW. (**a**) Summary of analysis results. The percentages of DEGs within each group are indicated in parenthesis. (**b**) A chart shows the percentage of DEGs in each category. (**c**) The Venn diagram shows the numbers of DEGs in each cellular component - nucleus, cytoplasm, mitochondrion and chloroplasts. The number in each intersection represents the mutually present DEGs in the corresponding cellular components. The information of DEGs in VMW is listed in Supplemental Tables S3, S4.

To determine the consequences of elevated OPDA in VMW and the potential cause of OPDA accumulation, the transcriptomic profiles of the two sectors were compared. Our previously established transcriptome using RNA-Seq technology on various types of tissues from ‘Marble Queen’ and its green reversion ‘Jade’^30^ was used as the reference sequences for current analysis. The RNAs from three paired VMW and VMG tissues were sequenced and a total of 143 and 101 million reads were obtained, respectively (Supplementary Table S1). DEG analysis showed that there were 850 DEGs (Fig. 3a). Among them, 52.5% were more abundant while 47.5% were less abundant in VMW than in VMG. There were 284 unique DEGs that could not be annotated (Supplementary Table S2) while 566 (~67%) could be annotated (Supplementary Tables S3, S4). To validate the RNA-Seq results, qRT-PCR analysis was employed to quantify the expression levels of some selected DEGs and un-differentially expressed genes. All selected genes involved in various biological processes had similar expression patterns between qRT-PCR and RNA-Seq analyses (Supplementary Table S5).

Among the 566 annotated DEGs, 306 were found to be more abundant (up) and 260 were less abundant (down) in VMW than in VMG (Fig. 3a). To facilitate the gene search and categorize their functions, further search for annotated DEGs matching Arabidopsis genes listed in TAIR v10 protein database were conducted. There were 60 DEGs in up and 71 in down lists with no known function. The remaining DEGs could be assigned to corresponding Arabidopsis Gene IDs (in ATG numbers) with known functions (Supplementary Tables S4, S5). Based on categorized functions, more than 21% up- and 11% down-regulated DEGs were involved in regulating transcription activities (Fig. 3b; Table 1). Close to 25% of down-regulated DEGs encode products of photosystem components (Fig. 3b). The Gene Ontology (GO) search based on cellular component and revealed by Venn diagram^37^ indicated that the most abundant (48 out of 170) DEGs in the up list were localized or functioned in the nucleus whereas the most abundant (36 out of 64) DEGs in the down list encode chloroplast proteins (Fig. 3c).

### OPDA plays an important role in reprogramming gene expression

OPDA, JA and JA-Ile are known to be involved in responses to biotic and abiotic stress as well as in plant growth and development^23^. However, unlike JA-Ile, OPDA is not perceived by the SCF^COI1^-JAZ-co-receptor complex^38^ and genes involved in the OPDA and JA signaling pathways are not identical^7^,^8^. OPDA has been shown that upon binding to the receptor CYP20-3, they form the cysteine synthase complex comprising serine acetyltransferase for cysteine synthesis. Increased cysteine can be metabolized into GSH to alter the redox state of the cells and to convey the redox signals that regulate expressions of OPDA-responsive genes (ORGs)^8^. Some of these reported ORGs in Arabidopsis could be transiently induced by OPDA treatment^7^, which might not all appear in our DEG list because of the different physiological status of variegated plants with constantly elevated levels of OPDA in color defective sectors as compared to OPDA-treated green plants. However, a certain number of observed ORGs^7^ involved in OPDA retrograde signaling network should be present in our DEG list if elevated OPDA plays any retrograde signaling role in VMW cells.

After comparing our DEGs with ORGs reported by Taki *et al*.^7^, we found that a significant proportion (19%) of Arabidopsis ORGs with annotation were transcription factors. Similarly, in our DEGs, a total of 73 (53 up- and 20 down-regulated) in VMW were also involved in modulating transcription activities (Table 1). This overrepresented group of DEGs (17% in DEG list with known functions) suggested that reprogramming gene expression might be a way of responding to the altered metabolism and ultimately benefit the white cells in dealing with the abnormal physiological status.

Among the up list DEGs, about 70% of them are stress-related transcription factors. Previously reported four groups of up-regulated transcription factors (AP2/EREB, WRKY, MYB, and Zinc-finger-type) induced by OPDA^7^ were all found in our up list (Table 1). They are known to be induced by biotic and abiotic stress for stress tolerance^39^,^40^,^41^,^42^. These stress induced transcription factors found in both normal green plants treated with OPDA in Taki *et al*.^7^ and current study in the VMW with elevated OPDA suggest that OPDA might function as a retrograde signaling molecule in VMW for modulating gene expressions to deal with stress conditions as proposed previously^7^,^23^.

Besides these four groups, there were additional four groups of transcription factors present in our up list also known to be stress-induced (Table 1), which were HSF-, HSP20-like-, bZIP- and NAC-type transcription factors^43^,^44^,^45^,^46^,^47^,^48^,^49^. These stress-related transcription factors could be specific transcription factors related to VMW cell physiological status.

### Increased activities of scavenging, small chaperones, and DNA repair in VMW Cells

**Figure 4:**
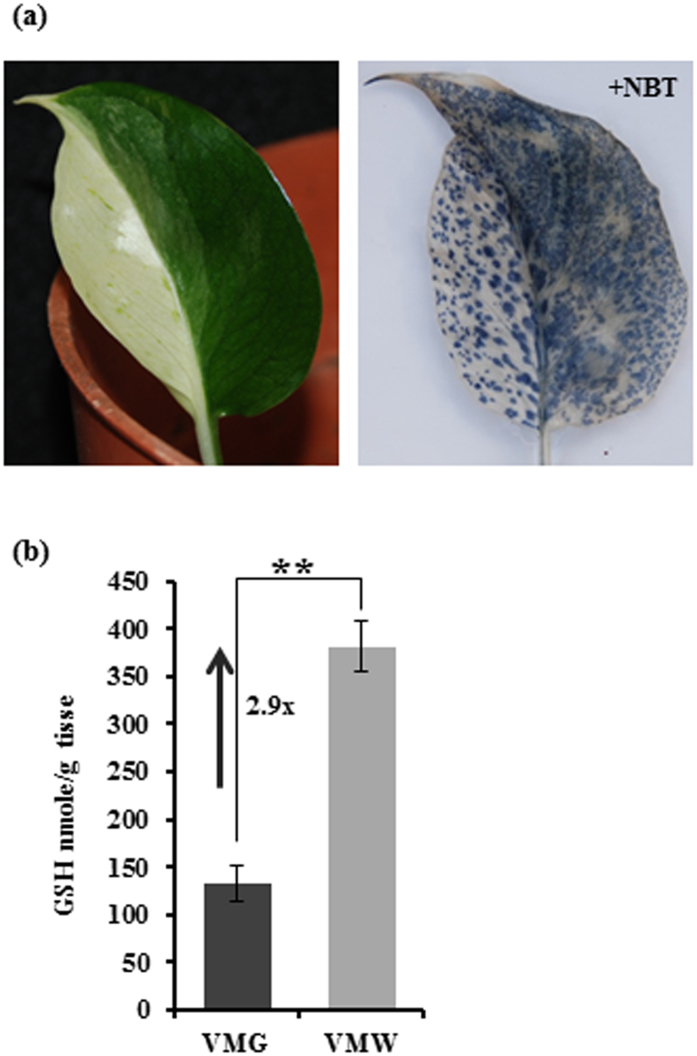
*In situ* detection of ROS and measurement of GSH contents. (**a**) A ‘Marble Queen’ leaf with distinct green and white sectors before (left) and after treatment with 6 mM NBT (right). The formation of insoluble dark blue diformazan came from the reduction of NBT by the presence of superoxide. (**b**) Total GSH was measured and compared between VMG and VMW. Data represents an average from three independent pairs of sectors ± SD. The arrow represents an increase (↑) in fold (x). **P < 0.01.

In addition to a group of factors identified in modulating transcription activity in VMW cells, a group of DEGs involved in ROS and nitric oxide (NO) scavenging activity and detoxification, small chaperones, and DNA repairing were uniquely presented in the up list (Fig. 3b; Table 2). About 32% showed induced levels of logFC ≥ 4 (Table 2). Scavenging activity is crucial for reducing oxidative stress from overproduction of ROS by electron transport^43^,^50^. ROS are mainly produced from chloroplasts and mitochondria, as well as in the endoplasmic reticulum during protein folding^43^. Among these up-regulated DEGs involved in scavenging activity, most are scavengers of ROS including glutathione *S*-transferases (GSTs), FERRITIN, immutans protein and dehydrogenase/oxidoreductase (Table 2). The overrepresented ROS scavengers in VMW suggested that VMW may have reduced levels of ROS compared to VMG. To determine whether it is the case, a fully expanded young leaf with distinct large areas of VMW and VMG was treated with NBT, which can form insoluble blue diformazan upon reduction by ROS^51^. The result showed less blue precipitation in VMW compared to VMG (Fig. 4a), indicating the ROS level in VMW was less than that in VMG.

Seven out of 16 (~44%) scavengers were GSTs (Table 2). It could be possible that the level of antioxidant GSH would be higher in VMW than VMG because GSH is a substrate of GST and participates in ascorbate-glutathione cycles to reduce ROS^52^,^53^. To confirm this speculation, the levels of GSH were measured and compared between VMW and VMG of the same leaf. GSH levels in VMW were found to be three-fold higher than in VMG (Fig. 4b). Elevated levels of GSH in VMW further support the observed results of reduced ROS in VMW (Fig. 4a).

In addition to reduced ROS levels in VMW, genes for protection against oxidative damage on DNAs and proteins were also found to be up-regulated in VMW (Table 2). There were twelve DEGs which encode heat shock proteins including six small heat shock proteins (Hsp20) known to be the first line of stress defense^54^ and three DnaJ, a universal chaperon induced by H_2_O_2_ across all kingdoms^55^. The increased expression of these genes could facilitate the removal of damaged and miss-folded proteins during ER stress. Genes responsible for DNA repairing (Rad21, Rad51 and Rad54) as well as those for DNA replication (topoisomerase and DNA polymerase subunit) and cell cycle control (CDC48) were also in the up list, indicating a potential lessening of the detrimental effects of damaged DNA during oxidative stress.

### Genes related to elevated OPDA in VMW

In order to understand how OPDA was accumulated in VMW, genes potentially involved in OPDA biosynthesis were carefully searched in our DEG list. Based on the Pfam domain analysis, we found that contig_8937 and_27131 (Supplementary Table S3) to patatin-like phospholipase family protein (AT1G61850) were up-regulated ~5-fold, whereas contig_5613 to mitochondria-localized class III phospholipase A1 (AT1G30370) and contig_13470 for phospholipase A2 (AT2G06925) were reduced 11- and 3-fold, respectively (Supplementary Table S4). The patatin-like phospholipase is involved in initial stage of JA biosynthesis by releasing its precursor fatty acid from thylakoid membrane lipids^56^. The latter two do not function in chloroplasts where OPDA biosynthesis pathway is localized^57^,^58^, so they may not be involved in the OPDA production. Therefore, the two up-regulated DEGs to patatin-like phospholipase could facilitate the generation of free fatty acids for OPDA production in VMW. In addition, two DEGs (contig_4904 and_10685) to alpha/beta hydrolases (AT5G17780 and AT4G36530) known to be responsible for fatty acid accumulation^59^ were also up-regulated ~3-fold (Supplementary Table S3). These genes might facilitate the production and accumulation of fatty acids in VMW for OPDA biosynthesis.

Among other DEGs involved in OPDA and JA biosynthesis pathways, we found that contig_5966 to OPDA reductase 3 (OPR3) was reduced 4-fold (Supplementary Table S4). In Arabidopsis, OPR3 is the most effective enzyme among OPDA reductases for converting OPDA to JA^60^,^61^. One of the consequences of low expression of *OPR3* in VMW could be the accumulation of OPDA. Indeed, Arabidopsis mutant *opr3* also accumulates OPDA^62^.

### Verified induced expression of potential ORGs in OPDA-treated PG plants

**Figure 5:**
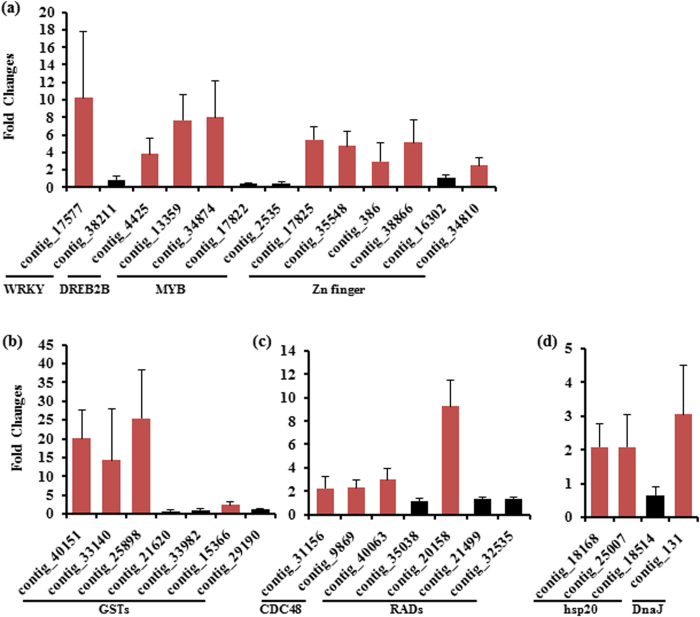
QRT-PCR analysis of selected DEGs in OPDA-treated PG plants. Selected DEGs listed in Table 1 for transcription factors (**a**), and Table 2 for scavenger GSTs (**b**), DNA replication and repair (**c**), and heat shock proteins (**d**) were quantified. Those induced by OPDA treatment greater than 2-fold were colored in red. Data represent the average of fold changes between treated and control (as 1) samples (n = three biological replicates) ± SD.

To examine whether above identified potential ORGs could be really induced by OPDA, some of them were selected and their expression levels in OPDA-treated PG plants were quantified by qRT-PCR. Compared to control-treated PG plants, qRT-PCR results show that nine out of 13 selected transcription factors were up-regulated greater than 2 folds (Fig. 5a). For those up-regulated genes in VMW involved in reducing ROS levels and ROS harmful effects, more than half of GSTs in up list (Table 2) were also induced more than two-fold (Fig. 5b). Additionally, both CDC48 and two out of five Rads (Fig. 5c) were induced by OPDA. Up-regulated DnaJ and two out of three most up-regulated Hsp20s in up list (Table 2) were also induced by OPDA treatment (Fig. 5d). These results indicate that majority of these potential ORGs could be induced by OPDA.

## Discussion

In this study, we used naturally variegated ‘Marble Queen’ to investigate potential survival strategies used by the white cells in the variegated leaves. Our analyses of elements and metabolites showed that there was no deficiency of elements in VMW compared to VMG, however the phytohormones and photosynthetic pigments were greatly reduced except for OPDA (Fig. 2 and Supplementary Fig. 1). Further comparing DEGs, we found several groups of ORGs that were up-regulated in VMW (Tables 1 and 2), and that their differential expression could benefit the survival of white cells. These genes include: (1) OPDA induced stress-related transcription factors reported in previous studies for stress tolerance; (2) ROS scavengers that correlate with elevated GSH and reduced ROS levels; and (3) protein chaperones and DNA repair genes for protecting cells from oxidative stress. We also discovered that the accumulated OPDA in the VMW may facilitate the reduction of ROS levels by increasing the production of GSH (Fig. 4) as well as the scavenging activity through up-regulating a group of GSTs (Table 2; Fig. 5b). Our results point toward the potential role of OPDA for maintaining white cell survival in variegated leaves as well as provide molecular bases as to why less ROS levels are in color defective sectors compared to neighboring green sectors as observed in our study of *E. aureum* and previous studies of Arabidopsis^16^,^17^. Additionally, comparative study of these special white and green sectors from the same leaf suggested that elevated OPDA plays an important role of dampening ROS-induced cytotoxicity under stress conditions.

Our transcriptome data reveal that major groups of genes which were up-regulated in VMW have functions involving in reducing oxidative stress to benefit white cell survival. The increased expression of stress-related transcription factors (Table 1) and genes encoding hallmark proteins for stress responses^63^, such as topoisomerase, Rad51, DnaJ, DnaK and GrpE (Table 2), indicate that white cells were under stress. Those up-regulated DEGs with scavenging activity in VMW (Table 2) could be the direct consequence of transcriptome reprogramming for dealing with stress as shown in *im*^15^ and *var2*^16^. Stress conditions are often accompanied by overproduction of ROS, such as O_2_^·−^ and H_2_O_2_. Thus, maintaining the ROS under a certain threshold to prevent accumulation to cytotoxic levels is an important survival strategy^22^. To cope with the excessive ROS, plants develop scavenging systems including scavenging enzymes and antioxidants to reduce ROS^43^,^50^. There are dozens of scavenging enzymes known in plants, such as superoxide dismutase, catalase, and glutathione peroxidase^64^, which are induced in mutants *im*^15^ and *var2*^16^. In our DEGs, only a group of genes for GSTs and one for a NO scavenger as well as some others for minor antioxidant enzymes, such as dehydrogenase/oxidoreductase, were found in the up list (Table 2; Supplementary Table S3). The GSTs are encoded by a large gene family^65^ that can use the antioxidant GSH as a substrate and participate in ascorbate-glutathione cycles to reduce ROS^52^,^53^. GST knockout mutant has found a positive correlation of GST expression levels with GSH concentration^66^. Indeed, we also found the VMW having ~3-fold higher GSH contents than that in VMG (Fig. 4b).

The reason for the increase of GSTs over the other scavenging enzymes in VMW is likely due to the close relationship between GSH and OPDA. The OPDA-induced retrograde signaling is thought to be mediated through the increase of cysteine biosynthesis and the conversion of newly produced cysteine to GSH, and ultimately convey the redox signal to the nucleus^8^. Thus, it is reasonable to deduce that elevated OPDA can result in increased GSH concentration. The elevated OPDA in VMW (Fig. 2b) associated with high GSH contents (Fig. 4b) agree well with the previous report of Park *et al*.^8^.

The present study and the others from Arabidopsis mutants *im*^15^ and *var2*^16^ have demonstrated that a suite of genes for reducing oxidative stress and protecting cells from oxidative damages were highly up-regulated in color defective sectors (white or yellow), suggesting that cells in the defective sectors experience more stress than green sector cells. But why are ROS levels in green sectors much higher than in the white sectors as documented in previous *var2* studies^16^,^17^ and this study (Fig. 4a)?

Our study with variegated Epipremnum probably provides a partial answer to this question. We have observed that OPDA levels were much high in VMW. Our speculation is that ROS in VMW cells were initially higher than the VMG cells. The high ROS may trigger and promote the lipid peroxidation to release OPDA precursors. This process would consume ROS and in turn reduce ROS levels. The increased OPDA would induce stress related genes to further reduce ROS levels by increasing scavenging activity via induction of a group of GSTs (Table 2). This is supported by the previous report that OPDA induced a subset of GSTs for increasing scavenging ROS^7^ as well as current study in OPDA-treated green plants (Fig. 5b). Since VMW and VMG are side by side in the same leaf, the processes of overproduction OPDA to quench the ROS would benefit the white cells in order to maintain their survival. Hence, we observed low ROS in VMW (Fig. 4a). In normal green plants, separated studies demonstrated that stress could transiently induce ROS^67^ and OPDA^24^,^25^. However, there is no report on the time-lag between stress-induced OPDA and ROS in the same experiment. Further research regarding exact conditions for ROS to trigger OPDA production is warranted.

As to the action mode, OPDA has been proposed to counteract JA effects in response to the increase of the singlet oxygen (^1^O_2_) on promoting programmed cell death^22^. Our current study also suggests that the elevated OPDA in the absence of JA could enhance scavenging activity and protect cells from oxidative stress. Regarding the perception of OPDA for triggering downstream signal cascade to modulate gene expressions, it has been shown that CYP20-3 could function as an OPDA receptor^8^, which are discussed in the following paragraph. The finding of JA-Ile rather than JA promoting the interaction of COI1 and JAZ1^38^ raises another possibility that OPDA isoleucine conjugate (OPDA-Ile) could be a bioactive form. A recent study by Floková *et al*.^68^ detected trace level of OPDA-Ile in wounded leaves of flowering Arabidopsis, which further opens a possibility that, like JA-Ile, OPDA-Ile is the active signaling component. A recent feeding experiment has also shown that OPDA-Ile has the potential to be bioactive^69^. In the current experimental system, whether Epipremnum plants have detectable levels of OPDA-Ile, and whether OPDA or OPDA-Ile is the active signal perceived in the young leaves of VMW and OPDA-treated green Epipremnum plants need further study.

OPDA is synthesized in chloroplasts and can be transported to cytosol and peroxisome; and it has been reported to function via its receptor CYP20-3, which is also localized in chloroplasts^8^. OPDA binding to its receptor triggers the phosphorylation on tyrosine. The downstream effects are the increase of cysteine production that in turn increases the concentration of thiol groups^70^. The high concentrations of cysteine and thiol groups often accompany accumulation of GSH^53^ which well agrees with our GSH results (Fig. 4b). Independent studies also showed that OPDA could induce GSH production^8^,^53^,^70^. Increased GSH has been proposed as a redox regulator of TGA (TGACG motif) transcription factors^8^,^71^ that ultimately modulate a subset of genes for stress tolerance^7^,^72^. Because the genome sequence information of ‘Marble Queen’ does not exist for the TGACG motif search, thus we were not able to determine whether any DEGs were under TGA transcription factor control at this time. Nevertheless, what appears to be the case in VMW is an elevation of OPDA (Fig. 2b) leading to an induction of stress-related transcription factors (Table 1), which were similar to ORGs reported by Taki *et al*.^7^ and supported by the results of the OPDA-treated green plants (Fig. 5), and further leading to increased GSH production (Fig. 4b) and scavenging activity. These OPDA-involved changes ultimately reduced ROS levels (Fig. 4a) and induced stress-related genes to benefit white cell survival.

OPDA production and accumulation in VMW might be mainly via enzymatic processes. Under normal physiological conditions, OPDA in plants is mainly derived from the fatty acid α-linolenic acid (18:3) which is present in monogalactosyl diacylglycerol (MGDG; 18:3/16:3) and digalactosyl diacylglycerol (DGDG; 18:3/16:0)^23^,^73^. MGDG and DGDG make up 85% of the glycoglycerolipids of the chloroplast thylakoid membrane^74^. Fatty acids are released from these glycolipids by the action of lipases DEFECTIVE IN ANTHER DEHISCENCE1 (DAD1, also called PLA1) and DONGLE, followed by lipoxygenases oxidation of α-linolenic acid before entering the JA biosynthesis pathway. This process is referred to as enzymatic oxidation for the production of OPDA from fatty acid precursors^73^,^75^. Although contigs similar to DAD1 and DONGLE could be found in our library, indicating that these genes were expressed, we did not observe any differential expressions of these contigs between VMW and VMG. Hence, reduced conversion of OPDA to JA resulting from low expression of *OPR3* and the high expressions of genes coding for patatin-like phospholipase for releasing the fatty acid from membrane lipids^56^ and alpha/beta hydrolases for facilitating the accumulation of fatty acids^59^ could be the keys for enzymatic accumulation of OPDA in VMW under our low light and unwounded conditions. In addition, non-enzymatic ROS mediated formation of oxylipins (oxygenated fatty acids) during various stress conditions have been observed^76^,^77^,^78^ and the production of OPDA from direct oxidation of α-linolenic acid has also been proposed^79^. The potential for non-enzymatic accumulation of OPDA in VMW from fatty acids directly released from MGDG and DGDG requires further study.

## Methods

### Plant materials

Variegated Epipremnum ‘Marble Queen’ plants were initially obtained from the University of Florida’s Mid-Florida Research and Education Center. They were grown in potting soil under glasshouse conditions at 23 °C and ~100 μmole m^−2^ s^−1^ light intensity. Fully expanded young leaves with distinct large areas of green and white tissues, which allow similar sizes of green and white sectors to be excised from the same leaf, were used for the comparative analyses. Green and white tissues from variegated leaves were harvested and pooled separately in liquid nitrogen. Then they were ground into powder and immediately aliquoted for acidic phytohormone, chlorophyll and carotenoid analyses as well as RNA isolation. Three pools of leaf sectors were used as three biological replicates.

### OPDA treatment

Green plants (PG) regenerated from green leaf sectors of ‘Golden Pothos’^27^ were maintained on MS medium in the growth chamber at 23 °C and ~100 μmole m^−2^ s^−1^ light intensity. For preparing OPDA solution, 1 mg of OPDA (Cayman Chemical Company, item number 88520) was dissolved in 1 ml of ethanol and then added into 100 ml of 0.5x MS salts to a final concentration of 30 μM^7^. A control solution was prepared by mixing 1 ml ethanol into 100 ml of 0.5x MS salts. After transferring to fresh medium for two weeks, plants with new-grown roots and leaves were first rinsed with 0.5x MS salts pH 5.6 to remove agar. Rinsed plants were placed in jars containing either OPDA or control solutions. After 24 h of treatment, young leaves were harvested for RNA isolation. Three individual plants per treatment were used as three replicates.

### Transmission electron microscopy (TEM) analysis

Green and white sectors with equal area were first excised from the same leaf (Fig. 1a) and then proceeded to further fine cutting, fixation and observation under a JEOL JEM 100 S transmission electron microscope following the procedure as previously reported in Hung *et al*.^27^.

### Measurement of Chls and carotenoids

To measure Chls and carotenoids in green and white sectors, the methods for extraction and quantification, and the equations for calculation were adopted from Lichtenthaler^80^. The contents of Chl*a* and Chl*b* were measured separately while two major groups of carotenoids: carotenes and xanthophylls were measured together.

### Acidic phytohormone analysis

Acidic phytohormones including ABA, SA, JA, JA-Ile and OPDA were analyzed using LC-MS/MS. Samples were processed and analyzed at the Proteomics Facility at the Donald Danforth Plant Science Center, St. Louis, MO, USA. To each ground sample, 900 μL of ice cold MeOH/ACN (1:1 v/v) and 10 μL of a 2.5 μM deuterium-labeled standard (d6-ABA, d4-SA and d2-JA) were added, and the samples were homogenized with the TissueLyser II (Qiagen) for 5 min at frequency of 15 Hz/sec, then centrifuged at 16,000 g for 10 min at 4 °C. The supernatants were transferred to new 2 mL tubes and the pellets were re-extracted as previously described. The second batch of supernatants was combined with the first and dried. The dried pellets were dissolved in 200 μL of 30% methanol, then centrifuged again to remove un-dissolved materials and the supernatants were transferred to vials for LC-MS/MS analysis. The injected volume of the sample was 50 μL. For LC separation, a monolithic C18 column (Onyx, 4.6 mm × 100 mm, Phenomenex) with a guard cartridge was used flowing at 1 mL/min. The LC system was interfaced with an AB Sciex 4000 QTRAP mass spectrometer equipped with a TurboIonSpray (TIS) electrospray ion source. The hormones ABA, SA, JA, JA-Ile, and OPDA were analyzed in negative ion mode using the following source parameters: curtain gas, 25 arbitrary units (a.u.); source gas 1, 50 a.u.; source gas 2, 50 a.u.; collision activated dissociation, high; interface heater, on; temperature, 550 °C; ion spray voltage, –4500. The LC gradient was from 60% solvent A (0.1% [v/v] acetic acid in Milli-Q water), to 100% solvent B (90% acetonitrile [v/v] with 0.1% acetic acid [v/v]) in 5 min. The LC was then returned to initial conditions in 1 min and re-equilibrated for both methods. Both quadruples (Q1 and Q3) were set to unit resolution. Analyst software (version 1.5) was used to control sample acquisition and data analysis. The 4000 QTRAP mass spectrometer was tuned and calibrated according to the manufacturer’s recommendations. All hormones were detected using MRM transitions that were previously optimized using a standard and a deuterium-labeled standard. For quantification, a series of standard samples containing different concentrations of unlabeled hormones was prepared. The peak area in samples was first normalized in the same way as used for the standard samples and then quantified according to the standard curve.

### Elemental analysis

For elemental analysis, fully expanded young leaves having distinct white sectors were used for harvesting green and white tissues. Pooled green or white tissues were subjected to elemental analysis as previously reported in Hung *et al*.^27^.

### Histochemical analysis of ROS

To detect the presence of ROS, nitroblue tetrazolium (NBT) (Roche) was used as a substrate that forms insoluble diformazan upon reduction. The method is adopted from Zhou *et al*.^51^ with some modification. A new fully expanded leaf was first photographed while attached to the plant without any touching. Then it was excised and quickly placed in a large syringe containing 6 mM NBT (in 10 mM Na-Citrate buffer, pH6) used to create the vacuum effect for infiltration until the leaf was fully submerged in the solution. The infiltrated leaf was kept in the dark for two hours at 25 °C to allow the formation of insoluble dark blue diformazan. The treated leaf was immersed in boiling 99% ethanol for 30 min so the pigments could be removed except the insoluble diformazan.

### Glutathione (GSH) analysis

The amount of GSH was measured using the Glutathione Assay kit (Sigma-Aldrich)^81^. About 0.15 g of leaf tissues ground into powder in liquid nitrogen was used to extract total GSH in 10 volumes of 5% 5-sulfosalicylic acid. The cell debris and precipitated proteins were removed by centrifugation at 10,000× g for 10 min at 4 °C. The collected supernatant was then used for assay following the manufacturer’s instruction.

### RNA-Seq and DEG analyses

Harvested tissues were first ground in liquid nitrogen, and used to isolate RNA with Qiagen RNeasy kit (Qiagen). DNase I treatment was applied to remove any DNA contamination. The RNA samples were sent to Genomic Sciences Laboratory at North Carolina State University for Illumina sequencing. The sequencing reactions were run on the Illumina GAIIx with single-end 72 bp read or on the Illumina Hyseq 2000 with single-end 100 bp read. The Consensus Assessment of Sequence and Variation (CASAVA) software (Illumina) was used to remove adaptor sequences, nucleotide library indexes and generate fastq files. The sequence data was summarized in Supplementary Table S1. For DEG analysis, the RNA-Seq reads were mapped to the *E. aureum* unique gene sequences from various types tissues of ‘Marble Queen’ and its green reversion ‘Jade’ in our previous study^30^ (NCBI Bioproject accession number PRJNA286034) by Bowtie^82^. The differential expression analysis was conducted by edgeR^83^ with the false discovery rate (FDR) < 0.05. Reciprocal blast^84^ using BLASTX and TBLASTN approaches was conducted to search the ortholog gene contigs matching to Arabidopsis genes listed at TAIR v10 protein database^85^ with a maximum E value of 1e-5 and the best hit was assigned.

### QRT-PCR analysis

The same RNA samples used for RNA-Seq analysis or RNA samples isolated from OPDA-treated and control-treated PG plants were used to generate first strand cDNA. The kits and procedures for performing qRT-PCR were the same as described in Hung *et al*.^27^. The calculation of Ct value was based on Pfaffl^86^. Data from three sets of biological samples were averaged. The information of primer sequences for specific genes is listed in Supplementary Table S6.

### Statistical analyses

All data were presented as means ± SD. Comparisons between VMG and VMW were performed using Student’s t-test. The asterisk indicates significant differences between two samples. Levels of statistical significance were set at *P < 0.05, **P < 0.01.

## Additional Information

**How to cite this article:** Sun, Y.-H. *et al*. Accumulation of high OPDA level correlates with reduced ROS and elevated GSH benefiting white cell survival in variegated leaves. *Sci. Rep.* **7**, 44158; doi: 10.1038/srep44158 (2017).

**Publisher's note:** Springer Nature remains neutral with regard to jurisdictional claims in published maps and institutional affiliations.

## Supplementary Material

Supplementary Information

Supplementary Table S2

Supplementary Table S3

Supplementary Table S4

## Figures and Tables

**Table 1:** List of DEGs involved in regulating transcription activities in VMW.

DEG code	BLAST hit	LogFC (VMG/VMW)	*P* value^a^
**VMW up** (**53 unique**)			
contig_17577	WRKY	−6.5	5E-17
contig_38211	WRKY5	−4.3	6E-11
contig_4425	DREB2B, AP2 domain containing protein	−2.4	8E-05
contig_10611	YABBY domain containing protein	−2.1	7E-03
contig_16550	Involved in oxygen sensing, AP2 domain containing protein	−3.4	6E-09
contig_36782	basic helix-loop-helix DND-binding domain containing protein	−1.6	4E-02
contig_21487	bZIP transcription factor	−3.2	7E-07
contig_17351	bZIP transcription factor	−3.2	1E-06
contig_8835	bZIP transcription factor	−3.1	4E-06
contig_23036	bZIP transcription factor domain containing protein	−1.7	2E-02
contig_30191	bZIP transcription factor domain containing protein	−1.7	3E-02
contig_23625	SCARECROW	−2.7	2E-05
contig_21753	SCARECROW	−2.3	1E-04
contig_31193	heat stress transcription factor	−2.7	8E-07
contig_36523	heat stress transcription factor	−2.6	4E-06
contig_13359	MYB family transcription factor	−2.6	3E-04
contig_34874	MYB family transcription factor	−2.5	5E-05
contig_17822	MYB family transcription factor	−2.0	1E-02
contig_2535	MYB family transcription factor	−1.5	4E-02
contig_35532	NAC domain protein	−4.4	3E-07
contig_32551	NAC domain protein	−4.3	4E-09
contig_39704	NAC domain protein	−4.1	2E-11
contig_26585	NAC domain protein	−3.9	1E-11
contig_30546	NAC domain protein	−2.1	1E-03
contig_25090	NAC domain protein	−1.9	4E-03
contig_38214	NAC domain protein	−1.6	3E-02
contig_3779	OVATE FAMILY PROTEIN 8	−3.6	1E-08
contig_35714	OFP13	−1.8	4E-02
contig_8475	PHD-finger family protein	−1.7	2E-02
contig_27359	PHD-finger family protein	−1.6	2E-02
contig_17825	CCT/B-box zinc finger protein	−3.8	9E-10
contig_35548	CCT/B-box zinc finger protein	−3.5	2E-09
contig_8747	GATA17-LIKE	−3.3	3E-07
contig_386	ZOS8-14 - C2H2 zinc finger protein	−5.7	2E-12
contig_38866	ZOS9-17 - C2H2 zinc finger protein	−1.8	2E-02
contig_16302	B-box zinc finger family protein	−1.7	3E-02
contig_5785	NF-X1-type zinc finger protein	−1.5	5E-02
contig_30249	B3 DNA binding domain containing protein	−2.1	2E-02
contig_2976	HSF-type DNA-binding domain containing protein	−1.8	7E-03
contig_4131	HSF-type DNA-binding domain containing protein	−1.8	9E-03
contig_34810	zinc finger, C3HC4 type domain containing protein	−2.0	2E-03
contig_3829	SUVR4, SET domain containing protein, histone methylation	−1.6	3E-02
contig_28844	pentatricopeptide repeat protein PPR986-12	−1.6	3E-02
contig_29472	SGS3, leafbladeless1, posttranscriptional gene silencing	−6.4	7E-23
contig_9433	SGS3, leafbladeless1, posttranscriptional gene silencing	−4.4	2E-13
contig_19647	SGS3, leafbladeless1, posttranscriptional gene silencing	−3.9	4E-13
contig_10221	HSP20-like, SGS domain containing protein	−1.8	1E-02
contig_2623	RNA-directed DNA polymerase	−2.3	2E-04
contig_2860	DCL2, dicer-like	−3.8	7E-11
contig_34277	DCL2, dicer-like	−3.5	4E-09
contig_39839	DCL2, dicer-like	−2.3	3E-04
contig_28197	ARGONAUTE 2	−2.5	9E-06
contig_7285	ARGONAUTE 2	−1.5	4E-02
**VMW down** (**20 unique**)			
contig_622	YABBY domain containing protein	2.5	5E-05
contig_30158	basic helix-loop-helix domain containing protein	3.8	1E-11
contig_17112	basic helix-loop-helix domain containing protein	3.5	1E-10
contig_4371	basic helix-loop-helix family protein	1.9	2E-02
contig_37907	basic helix-loop-helix family protein	1.7	4E-03
contig_26427	basic helix-loop-helix family protein	1.5	6E-03
contig_19235	GRAS family transcription factor domain containing protein	2.9	4E-07
contig_21537	MYB family transcription factor	2.4	5E-05
contig_40696	MYB family transcription factor	2.1	5E-04
contig_14213	myb-like DNA-binding domain containing protein	1.6	3E-02
contig_10943	transcription activator GLK1-like	2.7	4E-07
contig_29164	maturase K	2.7	2E-06
contig_37131	maturase K	2.6	3E-05
contig_310	tetratricopeptide repeat domain containing protein	2.4	3E-03
contig_9961	tetratricopeptide repeat domain containing protein	5.3	2E-17
contig_27106	FLOR1, transcription factor interacting protein	3.4	1E-08
contig_22855	FLOR1, transcription factor interacting protein	2.7	1E-06
contig_35148	HEMERA, PTAC12, plastid transcription	1.6	2E-02
contig_21887	RNA polymerase sigma factor	2.5	3E-06
contig_32589	NF-YC1, HAP5, core histone H2A/H2B/H3/H4	2.2	1E-03

^a^*P* value is 5% FDR corrected.

**Table 2:** List of DEGs involved in scavenging activity and protecting DNA and proteins that were up regulated in VMW.

DEG code	BLAST hit	LogFC (VMG/VMW)	*P* value^a^
**Scavenging activity** (**16**)			
contig_40151	glutathione S-transferase	−7.5	2E-27
contig_33140	glutathione S-transferase	−6.8	9E-26
contig_25898	glutathione S-transferase	−6.3	1E-23
contig_21620	glutathione S-transferase	−5.8	2E-14
contig_33982	glutathione S-transferase	−3.2	5E-08
contig_15366	glutathione S-transferase	−1.8	1E-02
contig_29190	microsomal glutathione S-transferase 3	−2.4	4E-04
contig_17179	PTOX, immutans protein	−6.0	4E-22
contig_24889	PTOX, immutans protein	−5.4	4E-19
contig_28806	non-symbiotic hemoglobin 2, responsible for NO scavenging	−7.6	3E-30
contig_29566	FERRITIN 3, essential to protect cells against oxidative damage	−1.8	5E-04
contig_26844	FERRITIN 4, essential to protect cells against oxidative damage	−1.6	8E-03
contig_21879	NAD(P)H dehydrogenase B3	−2.7	6E-06
contig_23663	short chain dehydrogenase/reductase family	−1.8	5E-02
contig_12947	short chain dehydrogenase/reductase family	−1.8	3E-02
contig_37841	short chain dehydrogenase/reductase family	−1.7	3E-02
**DNA activity** (**12**)			
contig_31156	CDC48, cell division control protein 48 homolog E	−1.6	3E-02
contig_9869	CDC48, cell division control protein 48 homolog E	−1.6	3E-02
contig_40063	RAD54, a SWI2/SNF2 family of DNA-stimulated ATPases	−4.0	9E-12
contig_31178	CID7, smr domain containing protein	−2.0	2E-03
contig_35038	DNA repair protein Rad51	−1.8	1E-02
contig_20158	SYN1, Rad21/Rec8 like protein	−4.0	6E-07
contig_21499	Rad21/Rec8 like protein	−2.0	1E-02
contig_32535	Rad21/Rec8 like protein	−2.4	6E-04
contig_35639	TOPBP1B – Similar to DNA replication protein TOPBP1	−3.6	2E-07
contig_15609	OsTOP6A2 – Topoisomerase 6 subunit A homolog 2	−1.9	2E-02
contig_35301	POLD2 – Putative DNA polymerase delta complex subunit	−1.8	1E-02
contig_8719	AtYLMG1, YGGT family protein	−2.9	2E-06
**Chaperone** (**13**)			
contig_18168	hsp20/alpha crystallin family protein	−6.2	5E-23
contig_25007	hsp20/alpha crystallin family protein	−4.7	3E-14
contig_18514	hsp20/alpha crystallin family protein	−4.1	1E-10
contig_38163	hsp20/alpha crystallin family protein	−2.9	6E-04
contig_31960	hsp20/alpha crystallin family protein	−2.4	2E-04
contig_9982	hsp20/alpha crystallin family protein	−2.3	3E-04
contig_34943	DnaK family protein	−2.3	1E-04
contig_8984	chaperone protein dnaJ-related drought-induced protein 1	−2.0	3E-03
contig_131	heat shock protein DnaJ	−7.2	1E-04
contig_35353	heat shock protein DnaJ	−1.7	1E-02
contig_7544	heat shock protein DnaJ	−1.5	4E-02
contig_10064	co-chaperone GrpE protein	−2.0	2E-03
contig_31187	co-chaperone GrpE protein	−1.5	4E-02

^a^*P* value is 5% FDR corrected.
