# Supplementary material for: Accumulation of high OPDA level correlates with reduced ROS and elevated GSH benefiting white cell survival in variegated leaves
Source: Sci Rep. 2017 Mar 9;7:44158. doi: 10.1038/srep44158 (PMC5343462; doi:10.1038/srep44158)
Supplement: Supplementary Information [file srep44158-s1.pdf]

Supplementary Information for

Accumulation of high OPDA level correlates with reduced ROS and elevated GSH benefiting white cell survival in variegated leaves

Ying-Hsuan Sun[1], Chiu-Yueh Hung[2], Jie Qiu[3], Jianjun Chen[4], Farooqahmed S. Kittur[2], Carla E. Oldham[2], Richard J. Henny[4], Kent O. Burkey[5], Longjiang Fan[3], Jiahua Xie[2,*]

*: Correspondence and requests for materials should be addressed to J.X. (jxie@nccu.edu).

This PDF file includes

Supplementary Figure S1

Supplementary Tables S1, S5 and S6

Other Supplementary data for this manuscript includes the following:

Supplementary Table S2. List of DEGs in VMW without annotation.

Supplementary Table S3. List of DEGs that were up-regulated in VMW with annotation.

Supplementary Table S4. List of DEGs that were down-regulated in VMW with annotation.

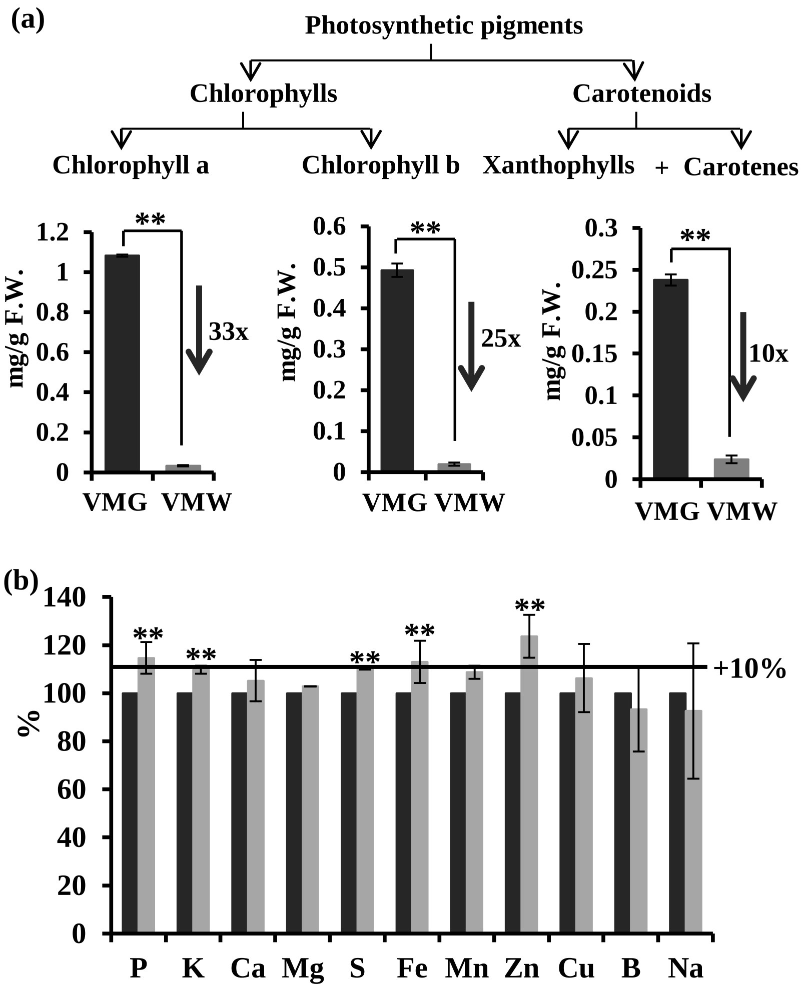

Supplementary Figure S1. Analysis of photosynthetic pigments and contents of elements in VMG (black) and VMW (gray). (a). Data represents an average from three independent pairs of sectors ± SD. The arrow represents a decrease (↓) in fold (x). F.W., fresh weight. (b). Data represents the average of percentage contents in VMW compared to that in VMG (as 100%) from three independent pairs of sectors ± SD. The line represents a 10% increase mark. **, P<0.01.

Supplementary Table S1. Summary of Illumina clean reads of VMG (M1G, M3G and M4G) and VMW (M1W, M3W and M4W) libraries.

Samples	Read counts	Total counts	Base in total [a]
M1G	37,187,711		
M3G	36,258,599	100,782,275	8,888,220,748
M4G	27,335,965		
M1W	34,696,599		
M3W	56,486,280	143,464,784	13,236,187,232
M4W	52,281,905		

[a]: M1G and M1W tissues were subjected to Illumina GAIIx 68 base single pass sequencing, whereas the rest of samples were subjected to Illumina Hyseq 2000, 100-base single pass sequencing.

Supplementary Table S5. Selected subset of DEGs confirmed by qRT-PCR[a].

		qRT-PCR	RNA-seq	
**Contigs**	**Functions**	FC[b] ±SD[c]	FC [b]	*p* value[d]
**VMW down**				
contig_138	cytochrome b6/f complex subunit IV	2.5±0.9	3.8	3E-03
contig_24619	photosystem I P700 chlorophyll a apoprotein A2	3.0±1.6	3.4	2E-03
contig_36747	ribulose-1,5-bisphosphate carboxylase/oxygenase large subunit	2.6±1.2	8.3	2E-08
contig_40765	photosystem II protein D1	3.9±2.2	6.4	1E-05
contig_30158	transcription factor bHLH61-like	16.4±6.2	13.5	1E-11
contig_1629	(R)-limonene synthase, putative	1.4±0.3	3.9	3E-03
contig_11989	(3S,6E)-nerolidol synthase 1	164.9±144.5	83.5	2E-26
**VMW up**				
contig_20302	secologanin synthase [Vitis vinifera]	-331.1±270.5	-43.0	5E-19
contig_8034	E3 ubiquitin-protein ligase RNF144A-like	-35.4±15.0	-23.3	1E-13
contig_28806	non-symbiotic hemoglobin, putative	-447.0±348.7	-199.2	3E-30
contig_29472	protein SUPPRESSOR OF GENE SILENCING 3-like	-338.3±306.7	-83.1	7E-23
contig_35342	manganese tracking factor for mitochondrial SOD2	-4.8±3.1	-3.3	8E-03
contig_37192	eukaryotic translation initiation factor 6-2 isoform 1	-6.5±2.3	-5.2	8E-05
contig_39704	transcription factor, putative	-36.0±11.7	-17.7	2E-11
**MEP pathway**				
contig_23274	1-deoxy-D-xylulose 5-phosphate synthase DXS1	2.7±0.7	2.5	3E-02
contig_23828	1-deoxy-D-xylulose 5-phosphate reductoisomerase	1.9±0.7	-1.1	1
contig_26347	2-C-methyl-D-erythritol 4-phosphate cytidyltransferase	1.6±0.3	-1.3	1
contig_35860	4-diphosphocytidyl-2C-methyl-d-erythritol kinase	1.7±0.3	-1.4	1

contig_10691	2-C-methyl-D-erythritol 2,4-cyclodiphosphate synthase	1.7±0.4	-1.2	1
contig_26392	1-hydroxy-2-methyl-2-(E)-butenyl 4-diphosphate synthase	1.1±0.4	1.4	1
contig_38115	1-hydroxy-2-methyl-2-(E)-butenyl 4-diphosphate reductase	0.9±0.6	1.9	0.4

[a]: Gene selection was based on their expression levels and each involved pathways. MEP pathway was selected as a representative of no differentially expressed except the first entry gene DXS1. [b]: FC, Fold changes comparing VMG to VMW. [c]: SD, standard deviation. [d]: *P* value is 5% FDR corrected.

Supplementary Table S6. Primer sequences for qRT-PCR.

**Contigs**	**Forward 5'-3'**	**Reverse 5'-3'**
contig_23274	GGAATGGCATAGGTGTTCCT	TGTCGTTCCAACACAGAAGCT
contig_10691	CCAAGCTGAGTCCCCACAA	GGCTCCGAGCAGATTGCATA
contig_23828	AATCCCGCGCAACCTACA	GTTGCTCGCCATCCAGATG
contig_26347	GGCAAAGGGAAACGGATGA	TTGGCCAAGGAGTGGAATG
contig_26392	ACAAAGCCACTGCCCAATTC	GTATGCGCCGGTTGTCAAT
contig_35860	TGACCATTCTTGCAGCTCCTT	CTGTGGGCTGCAAATCAATTT
contig_38115	CGTGAGGAAGCTTTGCAATTG	GCGCCATTGCTCATTTTAAGT
contig_20302	GTGGGTCACCGCATCAACA	CGTGTGGTCGATCTGTGGAA
contig_1629	AAGGAGATCCGCCCAGTCAT	AGGAAGAATGGCACCATCCA
contig_11989	GCGTGGGTGAGGAGGTTCT	GGCCTTAAGGTGCTTGTGAGA
contig_138	CGTAGGCTTAGCGGTCCTAGAA	CAAAGGAGTTGCAAATGGATCTG
contig_24619	CTACAGAAAAGTGGGCCAATCC	TAAGCCAGTGGCCCTTTCC
contig_36747	CCTGTTTCGGCCTGTGCTT	CGTTGGAGAGACCGTTTCGT
contig_40765	ATCAATCGGCCAAAATAACCAT	ACAGATTCGGTCAAGAGGAAGAA
contig_30158	GCTCCTCACGCATGCAATATC	CATCGAAGAATTGAAGCAAAAGG
contig_8034	AGTTCGGGTTCTGGAACATCTC	CGAACCTTCTGCGACATCTG
contig_28806	GCAAACCTGGTCACCTCGAA	GCGCCACCCACTTGAAGTA
contig_29472	AGGCCGTCGTCGGTTTCT	TCGTGCGTCATCGTGGTT
contig_35342	GGAGCTATTGATGAGGTGGGTTT	GCCAGCCCAGACGAGGTT
contig_37192	GGACCAGGCCACCTCTGTT	TTGCCGGAAATGTCATTGTC
contig_39704	CGCCCCATGGTTGTAAGG	CGCGGAGCTGGTGGATTA
contig_17577	GTCTCCGAGGTCCCTTATT	CACTGGCATCCATCACTAATC
contig_38211	CATCACTCTCTCTCTCTCTCTC	CCTTTAAGCCCTCCTCTTATC
contig_4425	CAGCACTTCCAACAACTCT	GAGTCTTCAACAGCCGATAC
contig_17825	GCGAGAAGTCGGTGTTG	AAGGTCGTCCATGCAAAG
contig_35548	CTTGTAGCGTGAGAGAACAG	TCCAGTGATGCAGGTAGT
contig_386	TGTTCCCTGTTCTTCCTTATTC	CTCTGATCCCAGTTGTTGTG
contig_38866	GCTGACATATGAGGCAGTAAA	GCTAGAAACCTAGTAAGCAGTC
contig_16302	GGAAACTGTCCCTCTGAATG	GCCTGAGGTACTGGTAGAA
contig_34810	CCTTGTCCCTCTCCTTCTT	CCTGGCTGGATGAACATAAC
contig_13359	TCAGCTCTCACACTCGAATA	CTCTGCTGGACGAACAATC
contig_34874	GAATTTCTGGAGGACCATACTT	GATGGGTCATGCTCTGTTTAT
contig_17822	CTGTTGTTGTCGCTCCATAG	TGCCTACCAAGAGGAGTTT
contig_2535	CAAGTGGTGAAGAGACTGAC	CTCTCTCTGGTCCTGTACTATT
contig_40151	CGGATTCTCCTCTTCGTTTAC	GACTCAGCCTTCTTGACATC

contig_33140	CTAGCGTTTGGGAGGAAATAA	ACCGTATGATACTCCACTCTAA
contig_25898	GGCACTCGGTACAGCTCCTT	CCACGCCTTTGAGCTTCAG
contig_21620	CGATTCGAGAGGGATGAAAG	GCGATGTGGATGCTGATAA
contig_33982	CCCTACTCGCCGCCTTCT	TCGGCCTGCTGTATACTTAATAATGA
contig_15366	GATGCCGGAGACGGAGAA	GCTGGGCTGCAACGTTTT
contig_29190	AGTGCTCCCCGTTGCTCTCT	GGGTTCCTACACCTACATCTGCAT
contig_31156	GGGCTGATGATGACGATTT	GGCTGTCCATCTGGTTATATG
contig_9869	CTGACTGAGATGGATGGAATG	CTCAGCAAAGCAGGATCTATAA
contig_40063	TGTCTTCCCTAGTCCCATATC	AAGAGAAGGTGTCCAGTTTATG
contig_35038	GAGTTCCTGGCATTGGTAAA	CCATAAAGCTCCCTTCTGTATC
contig_20158	ACCTCTCTCGGATACTCTTC	GGCAACTTTCATCCTCAAATAG
contig_21499	CTGAAGAGTTGCGGATGTATAG	TCCGTGTCCAGTCAGTTT
contig_32535	CATCTCCTCCACCATTGATAAG	CTGACAACACCCAGAAGAAG
contig_18168	TCTTCCTCTCACTGGATCTC	GGTGCGATAAGGAGAAGATAC
contig_25007	CTTCCTTGATCTCCGGTTATG	TATCAAGAGGAGAGGGAAGAC
contig_18514	GGATAGGAGAGAGGTGAAGAA	CCAACGCAGAAAGAACATAAG
contig_131	TCGACTGGTACCTCATTCTT	CTTGAGGGTGCTTGTTCTT
